# Medical Association of Georgia

**Published:** 1893-05

**Authors:** 


### MEDICAL ASSOCIATION OF GEORGIA.

The meeting at Americus was one of the largest in the history of the Association. There were some changes made in Constitution and By-Laws, which will be of great benefit to the Association in the future.

The nominating committee was done away with. We will have no more packed committees. Of late years the election of officers of the Association had assumed a political character to such an extent as to cause much unpleasantness and greatly impair the usefulness of the organization. To obviate this condition, Dr. W. F. Westmoreland moved as a substitute for Sections 11 and 15 of the By-Laws, that "all elections be held by ballot, *without nomination*, on the morning of the last day of the meeting." This was carried by a two-thirds vote.

Resolutions were also adopted, declaring the placing of professional cards in or about hotels, the calling attention through the secular press to the performance of operations, cures etc,, to be contrary to our ethics and not within keeping with the high tone to be expected of the members.

In his annual address the President took for his theme the establishment of a State Board of Health and a State Board of Examiners. He stated that in these matters Georgia was way behind her sister States, and that he looked forward with pleasure to the day when she would have a State Board of Health composed of the best medical talent of Georgia, and a Board of Examiners who would exclude quacks and charlatans from our borders.

The Association appointed a committee composed of one member from each congressional district to memorialize the legislature at its meeting in November next, and pledged itself as an Association and as individuals to heartily assist in the passage of a bill looking to the higher medical education and a State Board of Examiners.

The committee consisted of—

Third District—R. O. Engram, Chairman.

First District—Dr. R. J. Nunn.

Second District—Dr. T. M. McIntosh.

Fourth District—Dr. W. L. Bullard.

Fifth District—Dr. J. MacFadden Gaston.

Sixth District—Dr. Mark H. O'Daniel.

Seventh district—Dr. J. B. S. Holmes.

Eighth District—Dr. S. C. Benedict.

Ninth District—Dr. W. B. Tate.

Tenth District—Dr. E. C. Goodrich.

Eleventh District—Dr. J. A. Dunwoody.

Dr. R. E. Griggs, of Columbus, moved the appointment of a committee of one member of the Association from each Congressional district to urge members of the legislature to enact proper measures for the improvement of accommodations for the lunatic and inebriate asylum.

The President appointed on this committee the following members—

Fourth District—R. E. Griggs, Chairman.

First District—M. L. Boyd.

Second District—P. L. Hilsman.

Third District—J. D. Herrman.

Fifth District—W. F. Westmoreland.

Sixth District—K. P. Moore.

Seventh District—F. R. Calhoun.

Eighth District—John Gerdine.

Ninth District—T. D. McKowan.

Tenth District—H. B. McMaster.

Eleventh District—M. L. Currie.

The amendment to the Constitution, which provided for the election of lady physicians, graduates of the *regular schools*, was adopted.

We endorse the action of the Association, and earnestly hope that at our next meeting there will be a large number of new members from this source.

The election of Dr. Elliott as President, demonstrated that in this instance the office sought the man instead of the man seeking the office.

In electing Dr. Elliott, the Association has done itself great honor, and we anticipate that his administration will bring our Association to that high standard of excellence which it justly deserves. The unanimous election of the President showed that the politicians and their dark and mysterious ways have been forever crushed, which we know will meet with the strongest endorsement of the profession of Georgia.

Our next meeting will be held in Atlanta next April, and we extend to the profession of the State a hearty welcome.

The banquet tendered the Association by the physicians and citizens of Americus the previous evening was largely attended, the dining room of the Windsor Hotel being taxed to its utmost capacity. Dr. Dan H. Howell, of Atlanta, acted as Toastmaster.

The following is a list of the toasts and speakers :

"The Medical Association of Georgia," by A. A. Smith, of Hawkinsville.

"Professional Courtesy," by Dr. K. P. Moore, of Macon.

"City of Americus," by Mr. George D. Wheatley.

"The Triumphs of Modern Surgery," by Dr. J. MacFadden Gaston.

"The Law," by Judge W. B. Guerry.

"The Physician Himself," by Dr. F. W. McRae.

"The Press," by Dr. W. P. Burt.

"Woman," by Dr. R. E. L. Barnum.

"Our Country," by Hon. Chas F. Crisp.

"Our Host," by Dr. T. M. McIntosh.

### CLIMATE AND CONSUMPTION.

The Congress of Medico-Climatology will convene in the Art Building in Chicago May 29th, continuing one week. A choice programme has been arranged and many of the most noted climatologists have promised to be present. This Congress promises to be one of the most interesting that will meet during the World's Fair year. Thursday, June 1st, has been appointed a Field Day for the discussion of the causative and curative relations of Climates to Consumption. Reports from all parts of the world will be presented. All physicians are cordially invited to be present.

JOHN A. ROBINSON, M. D.,
T. C. DUNCAN, M. D.,
L. B. HAYMAN, M. D.,
Programme Committee.

---

Dr. J. B. S. Holmes, of Rome, has leased several residences for the accommodation of patients until the completion of his sanitarium, which will be about November 1st.

As is well known, his sanitarium was completely destroyed by fire last January. The new building will be a model of elegance and comfort.

---

The forty-fourth annual session of the American Medical Association will be held in Milwaukee, Wis., June 6, 7, 8 and 9, commencing on Tuesday, at 11 a. m.

---

A CENTENARIAN PHYSICIAN.—Dr. De Bossy, of Havre, has reached his one hundredth year. He is still in practice, and has been awarded a medal for his conduct during the last cholera epidemic. He was present at a dinner given a few days afio in honor of his one hundredth birthday. In a speech which he made on the occasion he said his father had lived to be one hundred and seven years of age, and ne hoped to do the same.

